# Vicious cycle of oxidative stress and neuroinflammation in pathophysiology of chronic vascular encephalopathy

**DOI:** 10.3389/fphys.2024.1443604

**Published:** 2024-08-05

**Authors:** Tetiana R. Dmytriv, Khrystyna V. Duve, Kenneth B. Storey, Volodymyr I. Lushchak

**Affiliations:** ^1^ Department of Biochemistry and Biotechnology, Vasyl Stefanyk Precarpathian National University, Ivano-Frankivsk, Ukraine; ^2^ Research and Development University, Ivano-Frankivsk, Ukraine; ^3^ Department of Neurology, I. Horbachevsky Ternopil National Medical University, Ternopil, Ukraine

**Keywords:** ROS, DAMPs, microglia, NF-κB, inflammasome, vicious cycle

## Abstract

Chronic vascular encephalopathy (CVE) is a frequent cause of vascular mild cognitive impairment and dementia, which significantly worsens the quality of life, especially in the elderly population. CVE is a result of chronic cerebral hypoperfusion, characterized by prolonged limited blood flow to the brain. This causes insufficient oxygenation of the brain leading to hypoxia. The latter can trigger a series of events associated with the development of oxidative/reductive stresses and neuroinflammation. Addressing the gap in knowledge regarding oxidative and reductive stresses in the development of vascular disorders and neuroinflammation can give a start to new directions of research in the context of CVE. In this review, we consider the hypoxia-induced molecular challenges involved in the pathophysiology of CVE, focusing on oxidative stress and neuroinflammation, which are combined in a vicious cycle of neurodegeneration. We also briefly describe therapeutic approaches to the treatment of CVE and outline the prospects for the use of sulforaphane, an isothiocyanate common in cruciferous plants, and vitamin D to break the vicious cycle and alleviate the cognitive impairments characteristic of patients with CVE.

## 1 Introduction

Scientific data suggests that more than 16% of individuals over the age of 60 suffer from moderate to severe cognitive disorders caused by cerebrovascular diseases (Owolabi et al., 2018). In particular, vascular encephalopathy is increasingly recognized as the cause of such symptoms as inappropriate behavior, decreased motivation, memory defects, coordination problems, and others. These brain disorders are associated with a violation of brain blood supply leading to the development of cerebral hypoperfusion, hypoxia, ischemia, and reoxygenation, with functional consequences for the brain. Long-term chronic low perfusion in the whole brain or local brain regions causes chronic vascular encephalopathy (CVE). Depending on the severity of the impairment, this is divided into mild vascular cognitive impairment and vascular dementia (Jellinger, 2013). The causes of these problems are varied and complex, making the pathogenetic mechanisms underlying the disease controversial. Dysregulation of cerebral blood flow, resulting in inadequate blood supply to the brain is one common factor among all of these causes (Yu et al., 2022). As a result, the impaired blood supply to the brain disrupts memory, cognition, and behavior, affecting neural networks and their functionality (Jellinger, 2013).

Inflammation and oxidative stress are among the key factors in the pathophysiology of vascular cognitive impairment and dementia. Brain hypoxia and ischemia, caused by chronic cerebral hypoperfusion in CVE, are fundamental factors that induce a series of pathological processes, including induction of oxidative stress and activation of the resident immune cells in the brain. In turn, this contributes to the development of neuroinflammation to maintain the stability of the microenvironment. However, excessive activation of the inflammatory response can cause significant damage to the brain by reactive oxygen species (ROS) due to their enhanced generation and contribute in this way to neurological dysfunction (Tian et al., 2022). Neuroinflammation is usually tightly connected with the development of oxidative stress. This results from an imbalance between ROS generation and elimination in favor of the first with various physiological consequences (Lushchak and Storey, 2021). ROS cause numerous oxidative modifications of biomolecules, including oxidation of proteins, lipids and DNA, that eventually lead to apoptotic death, contributing to the progression of cognitive impairment (Poh et al., 2022; Butterfield, 2023; Dmytriv et al., 2023).

**FIGURE 1:**
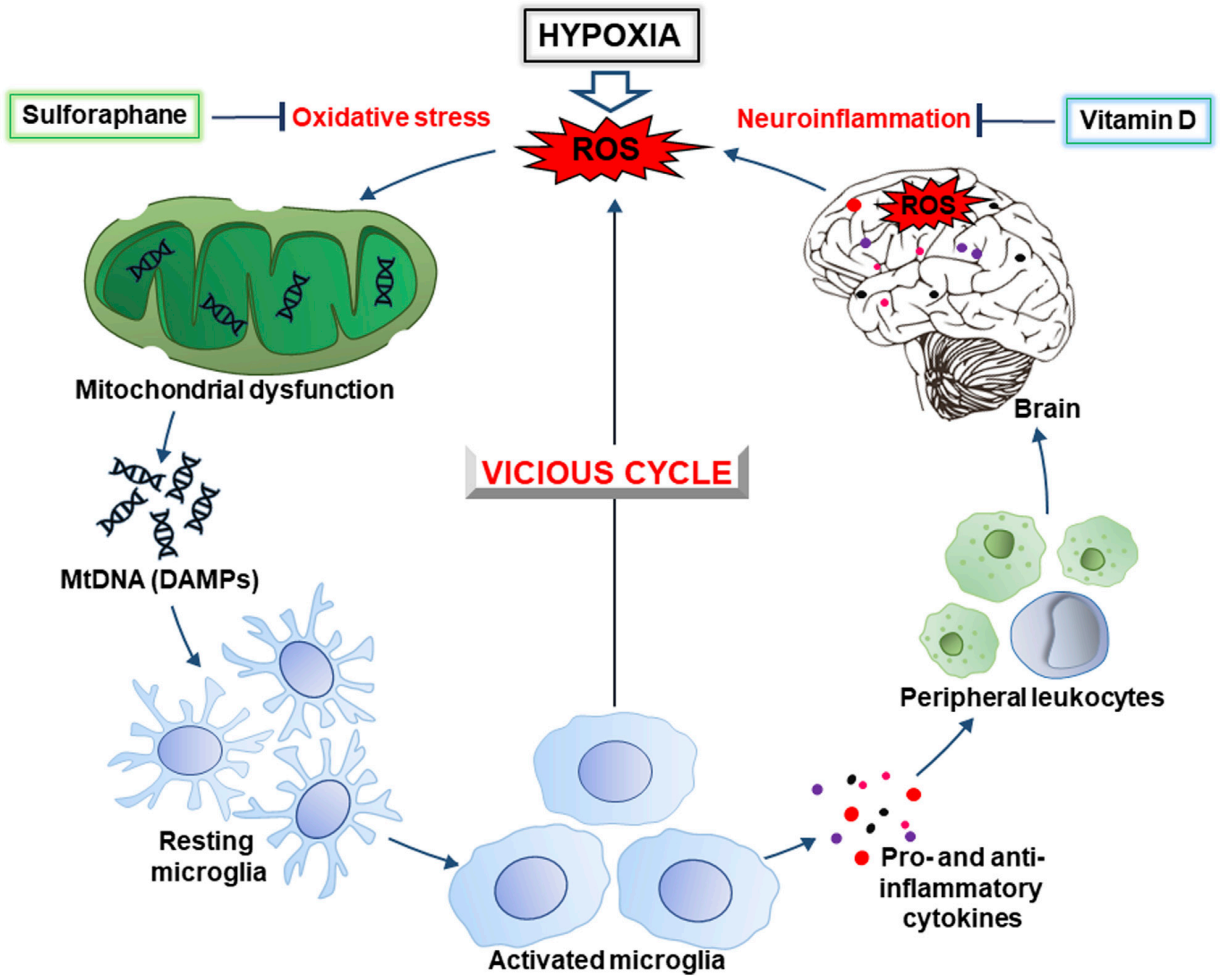
The hypoxia-inducible vicious cycle of neurodegeneration. Lack of oxygen during hypoxia causes increased generation of ROS (reactive oxygen species) due to excessive escape of electrons from the mitochondrial electron-transport chain. In turn, this causes the development of oxidative stress and mitochondrial dysfunction, as well as the release of DAMPs (damage-associated molecular patterns), in particular, mitochondrial DNA (mtDNA). Ultimately, this leads to activation of the brain’s resident immune cells, the microglia. The latter, depending on the conditions, produces pro- and anti-inflammatory cytokines, ROS, as well as chemokines that contribute to the recruitment of peripheral leukocytes. In general, this causes the development of neuroinflammation accompanied by an even greater generation of ROS resulting in intensification of oxidative stress. This creates a vicious cycle of oxidative stress and inflammation that contributes to neurodegeneration. Sulforaphane and vitamin D, that inhibit the development of oxidative stress and inflammation, may be promising drugs to break this cycle and prevent cognitive impairment.

Various substances of natural origin can potentially be used for the prevention and relief of neuroinflammation and oxidative stress in CVE. For example, sulforaphane, an isothiocyanate from cruciferous vegetables, is a promising neuroprotectant that prevents and alleviates the symptoms of many neurological disorders (Klomparens and Ding, 2019). In addition, vitamin D may also be promising in this direction. In particular, vitamin D receptor signaling in microglia inhibits neuroinflammation (Cui et al., 2023). Understanding the molecular mechanisms of the above-mentioned pathological processes will provide a valuable theoretical basis for the prevention and treatment of CVE. This review aims to shed light on oxidative and reductive stresses induced by hypoxia and reoxygenation and the development of neuroinflammation in CVE. In particular, we combine oxidative stress and neuroinflammation into a vicious cycle of neurodegeneration (Figure 1) that may underlie the pathophysiology of CVE and outline the potential of using sulforaphane and vitamin D to break this vicious cycle.

## 2 Hypoxia-inducible challenges in chronic vascular encephalopathy: focusing on oxidative stress

Hypoxia is one of the most important features of CVE and is much more dangerous when combined with reoxygenation (the so-called “oxygen paradox”). There are several mechanisms for developing hypoxia and related vascular dysfunctions and compromised oxygen delivery to the brain (Dhillon et al., 2022). Below we list some of the key ways in which hypoxia can be developed in the context of CVE:

1. Vascular insufficiency caused by chronic vascular changes, such as stenosis (narrowing) or occlusion of blood vessels supplying the brain, which can lead to reduced blood flow and oxygen delivery (cerebral hypoperfusion).
2. Systemic dysfunction of endothelial cells lining blood vessels. Endothelial cells perform several important functions: (*i*) regulation of vascular tone, (*ii*) control of blood flow and clotting, and (*iii*) production of cytokines and adhesion molecules that regulate and direct the inflammatory process. Under homeostatic conditions, the endothelium maintains normal vascular tone and blood flow, and pro-inflammatory factors are practically absent. However, numerous risk factors such as hyperglycemia, hypercholesterolemia, hypertension, smoking, obesity, and others can initiate a chronic inflammatory process, that is accompanied by a loss of vasodilator and antithrombotic factors and an increase in vasoconstrictors and prothrombotic products, contributing to cardiovascular diseases (Widlansky et al., 2003). The presence of risk factors for cardiovascular diseases with subsequent damage to cerebral vessels is associated with an increased risk of developing cognitive impairment in elderly patients, and increased levels of markers of endothelial damage are associated with an increased risk of developing vascular dementia (Quinn et al., 2011; Sabayan et al., 2014). The latter emphasizes the importance of endothelial dysfunction in the pathophysiology of CVE.
3. Microvascular disease related to damage of small vessels, capillaries, and arterioles in the brain. These disrupt the microcirculation of blood in the brain.
4. Ischemic events such as transient ischemic attacks or small ischemic strokes may temporarily or permanently disrupt blood flow to specific brain regions.
5. Chronic inflammation may cause vascular dysfunction and hypoxia. This impairs blood vessel function, compromises the blood-brain barrier (BBB), and exacerbates vascular pathology.
6. BBB disruption that may also compromise the entry of substances, including inflammatory cells and molecules, into the brain, that can cause vascular dysfunction.
7. Mitochondrial dysfunction leading to impaired cellular energy production and the development of oxidative stress contributing to vascular damage.
8. Increased production of reactive oxygen species (ROS), for example, by xanthine oxidase (XO), may lead to vascular dysfunction. XO, together with xanthine dehydrogenase (XDH), are interconverting isoforms of the same enzyme known as xanthine oxidoreductase (XOR). XDH catalyzes the conversion of xanthine and hypoxanthine into uric acid producing NADH, whereas XO catalyzes the same reaction producing ROS such as superoxide anions and hydrogen peroxide as byproducts (Houston et al., 1999; Burrage et al., 2023). In addition, XO secondarily leads to peroxynitrite formation, a highly reactive compound produced by the reaction of nitric oxide and superoxide radicals, that is considered to be a marker of oxidative and nitrosative stresses. Under physiological conditions, XOR is mainly found in the dehydrogenase form, whereas in inflammatory situations XOR is usually found as XO (Sabán-Ruiz et al., 2013). The latter has a high affinity for glycosaminoglycans on the vascular endothelium, where its immobilization induces endothelial dysfunction via ROS production (Houston et al., 1999; Burrage et al., 2023), that can potentially lead to hypoxia.

**FIGURE 2:**
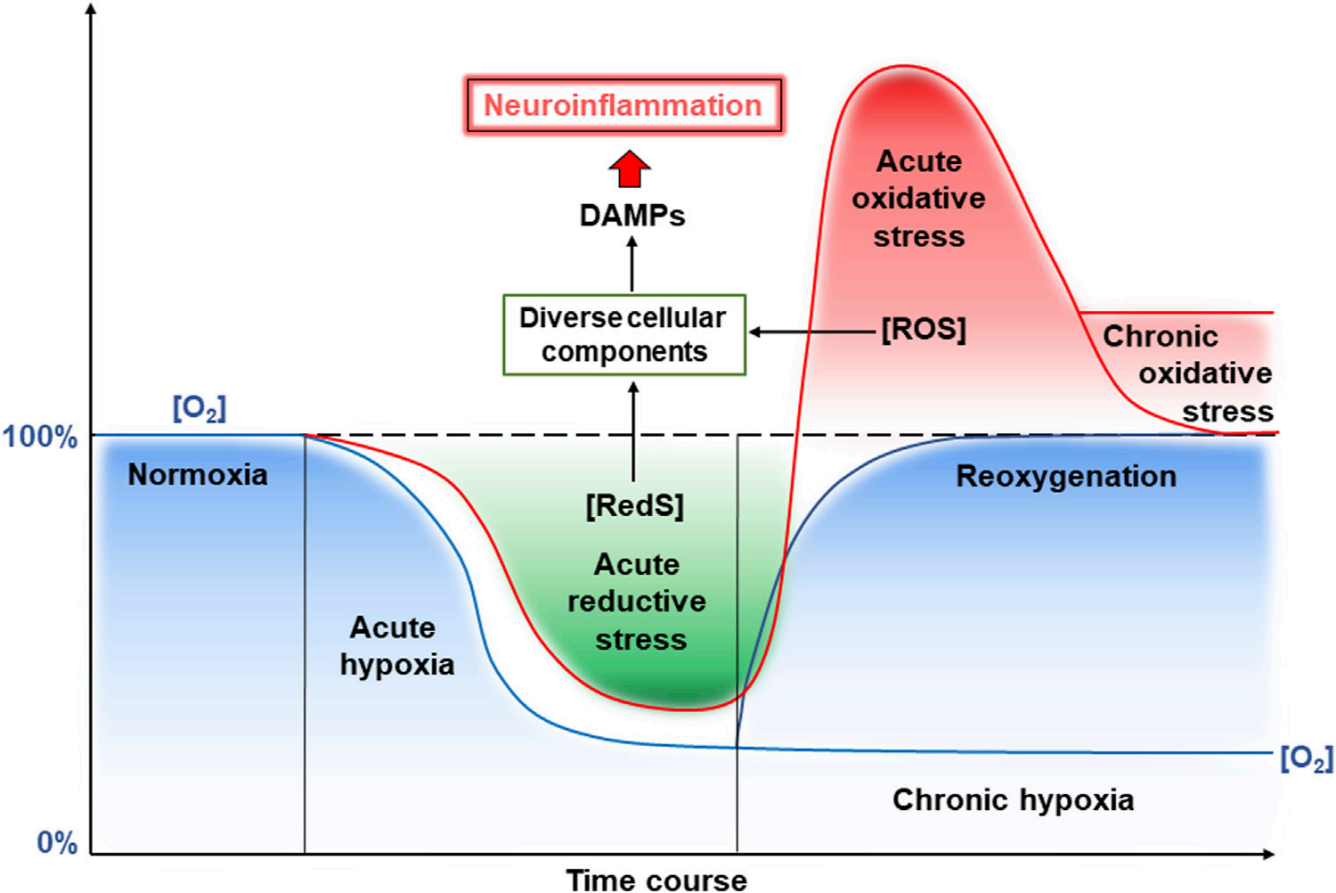
Reductive and oxidative stresses and neuroinflammation induced by hypoxia and reoxygenation. See a detailed description in the text. Abbreviations: RedS – reductive substrates, ROS – reactive oxygen species.

Overall, the development of hypoxia in CVE involves a combination of vascular, inflammatory, and metabolic abnormalities disrupting oxygen delivery and its utilization by the brain, contributing to neuronal dysfunction and cognitive impairment. Short-term hypoxia is called acute hypoxia, whereas prolonged oxygen limitation is called chronic hypoxia (Figure 2). As mentioned above, a hypoxic period may be changed to normoxia by restoration of blood flow that can result from the opening of occluded or narrowed vessels. This leads to reoxygenation and also frequently results in damage to vessel walls. This is due to enhanced ROS generation and the whole complex of events with damaging consequences has been called “oxygen paradox”. Now we will consider molecular events induced by hypoxia and reoxygenation that are related to brain damage due to ROS and induction of inflammation, termed neuroinflammation, since it occurs in the brain (Figure 2). Reduced oxygen levels under hypoxia not only lead to decreased production of ATP in mitochondria but also increase the levels of electrons in the electron transport chain of mitochondria. This state where the reductivity of intracellular space is increased has been called “reductive stress” (Lushchak, 2011). Excess “reductive force” or reducing substances (e.g., RedS) disrupts many cellular processes particularly due to the interaction of reducing equivalents with various cellular components (proteins, nucleic acids, etc.) leading to the production of so-called damage-associated molecular patterns (DAMPs).

Hypoxic conditions can also lead to mitochondrial dysfunction, with impaired operation of the electron transport chain (ETC) and ATP production. In certain cases, hypoxia-induced dysfunction can enhance the generation of ROS causing various forms of damage. These processes lead to the production of mitochondrial-derived DAMPs, mainly ROS-modified mitochondrial DNA (mtDNA) and mitochondrial proteins (Dmytriv et al., 2023). These DAMPs may be released from cells and trigger the production of proinflammatory cytokines, thereby contributing to oxidative stress. The latter is an imbalance between oxidants and antioxidants in living organisms in favor of OXIDANTS with certain changes in physiological processes (Lushchak, 2014). This imbalance can occur due to decreased oxygen availability (hypoxia), or impairing the function of oxygen-related antioxidant enzymes such as superoxide dismutase and catalase. Hence, reductive stress also can cause tissue damage and activate inflammatory pathways via the production of DAMPs. The stress induced by hypoxia is accompanied by damage that leads to the activation of proinflammatory pathways due to production of proinflammatory cytokines, chemokines, and other proinflammatory mediators. Again, the inflammatory processes may also contribute to the production of DAMPs from damaged cell constituents, amplifying reductive stress through activation of redox-sensitive signaling pathways. Collectively, these events form a vicious cycle of worsening hypoxia-induced damage (Figure 1).

Hypoxia can also induce endoplasmic reticulum stress due to the disruption of protein folding and leads to the accumulation of misfolded proteins (Díaz-Bulnes et al., 2020). The latter, together with heat shock proteins (HSPs) and high-mobility group box 1 (HMGB1) protein, may operate as DAMPs, contributing further to inflammation and reductive stress. If hypoxia is prolonged or becomes more severe, this can result in cell death via apoptosis or necrosis. Released intracellular components and DAMPs further contribute to tissue damage via exacerbating inflammatory and reductive stress responses associated with hypoxia.

To this end, hypoxia-induced stress production of DAMPs may be enhanced due to activation of glycolysis and production of such side products as methylglyoxal (Garaschuk et al., 2018). However, this can worsen the situation due to the induction of reductive stress that can enhance CVE severity via provoking mitochondrial dysfunction, proinflammatory responses, endoplasmic reticulum stress, and cellular damage (Semchyshyn, 2021). Understanding these molecular mechanisms may be useful in developing targeted therapeutic strategies to mitigate the detrimental effects of hypoxia and oxidative/reductive stress in CVE.

Hypoxia-induced temporary partial or full closure of blood vessels may be crucially changed by opening the vascular lumen. However, the expected potential positive result is, in fact, the opposite, because this substantially enhances the generation of ROS and causes massive damage to tissues. Such an effect of reoxygenation has been called the “oxygen paradox” (Hearse et al., 1978). Generally, the oxygen paradox defines a phenomenon where reoxygenation (restoration of oxygen supply) following a period of hypoxia or ischemia can lead to a substantial increase of ROS generation and induction of oxidative stress leading to tissue damage (Lushchak, 2011). There are several reasons for such a “paradoxical response” that can occur under various conditions, such as during organ transplantation, or ischemia-reperfusion injury in the brain or other organs. Such processes is believed to contribute the most to damage to the brain at reoxygenation.

As mentioned above, under hypoxic conditions, a highly reduced state of the mitochondrial ETC can develop and is called reductive stress (Lushchak, 2011). During reoxygenation, “excessive electrons” escape the mitochondrial ETC and interact with molecular oxygen due to increased oxygen availability. This gives rise to a burst of ROS production. Furthermore, ROS such as superoxide anion radicals, hydrogen peroxide, and hydroxyl radicals, can attack cellular components causing oxidative damage to macromolecules including lipids, proteins, and DNA. These processes are augmented by the accompanied disruption of mitochondrial function related to ETC dysfunction, also resulting in increased ROS production within mitochondria. ROS migration out of the mitochondria will then contribute to cell damage. Hence, mitochondrial dysfunction further exacerbates oxidative stress and contributes to cell death pathways.

Both pathways described in the previous paragraph collectively trigger inflammatory responses, activating immune cells and the release of pro-inflammatory cytokines and chemokines. In the brain, under these conditions, microglia are activated (Dmytriv et al., 2023). Inflammation amplifies oxidative stress by promoting ROS generation and impairing antioxidant defenses and, together, they form a vicious cycle that worsens the situation (Figure 1).

Homeostasis of calcium is also substantially challenged during reoxygenation (Hearse et al., 1978). Induction of oxidative stress and inflammation can lead to intracellular calcium overload, disruption of calcium homeostasis, and activation of calcium-dependent enzymes, such as phospholipases and proteases (Mattson, 2007). Again, calcium overload may contribute to cell death pathways and oxidative stress and in some way be incorporated into the above-mentioned vicious cycles.

Reactive nitrogen species (RNS) are also important players in reoxygenation. Nitric oxide (^•^NO) is very important for blood vessel operation because it causes vasodilation. During reoxygenation, ^•^NO production can be enhanced and this can improve blood supply to the brain due to vasodilation (Li and Jackson, 2002). However, ^•^NO is a free radical that can interact with many components in the brain. Moreover, at high levels, ^•^NO can interact with superoxide anion radicals (O_2_^•-^), that are plentiful during reoxygenation and form peroxynitrite, a highly reactive molecule that causes oxidative damage to biomolecules and exacerbates tissue injury (Lushchak and Lushchak, 2021). Finally, ROS and RNS formed during reoxygenation in excessive amounts may activate redox-sensitive signaling pathways, such as nuclear factor kappa B (NF-κB) and mitogen-activated protein kinase (MAPK) pathways (Kruk et al., 2019). Collectively, these regulate inflammatory and cell survival/death responses and dysregulation of these pathways contributes to oxidative stress and brain damage at CVE.

Hence, hypoxia followed by reoxygenation induces intensive oxidative stress via a complex interplay between oxygen availability, ROS and RNS generation, inflammatory responses, and cellular signaling pathways. Strategies to mitigate the fluctuations in redox processes may involve antioxidant therapies, modulation of inflammatory responses, and preservation of mitochondrial function to alleviate damage and improve tissue outcomes.

## 3 Neuroinflammation in chronic vascular encephalopathy

Inflammation is a protective response of the immune system that acts in response to aseptic injury, for example, sterile surgical interventions and non-aseptic interventions (viral or bacterial invasion). Activation of the inflammatory response leads to the migration of tissue leukocytes, in particular, monocytes, to the site of inflammation where they differentiate into macrophages, providing phagocytosis and control of the local microenvironment. Although inflammation is essentially a beneficial defense mechanism, an excessive pro-inflammatory response causes tissue damage and has negative functional consequences (Lyman et al., 2014).

Several experimental studies have shown a relationship between neuroinflammation and cognitive disorders (Sun et al., 2015; Tan et al., 2015; Tian et al., 2015; Hajiluian et al., 2017). In particular, chronic neuroinflammation is observed in patients suffering from vascular cognitive impairment at various stages of vascular dementia, including pre-clinical, clinical, and severe stages (Poh et al., 2022). Animal studies show that chronic cerebral hypoperfusion, that is a key factor of CVE, can cause short- and long-term neuroinflammation, damaging the myelin sheath, the BBB, and the grey matter (Liu et al., 2013; Miyanohara et al., 2018; Poh et al., 2021). Therefore, neuroinflammation appears to play a significant role in cognitive decline. Typically, it includes features such as activation of microglia, resident immune cells of the central nervous system (CNS), increased levels of proinflammatory cytokines and chemokines, recruitment of peripheral immune cells, and local brain damage (Moyse et al., 2022; Tian et al., 2022).

**FIGURE 3:**
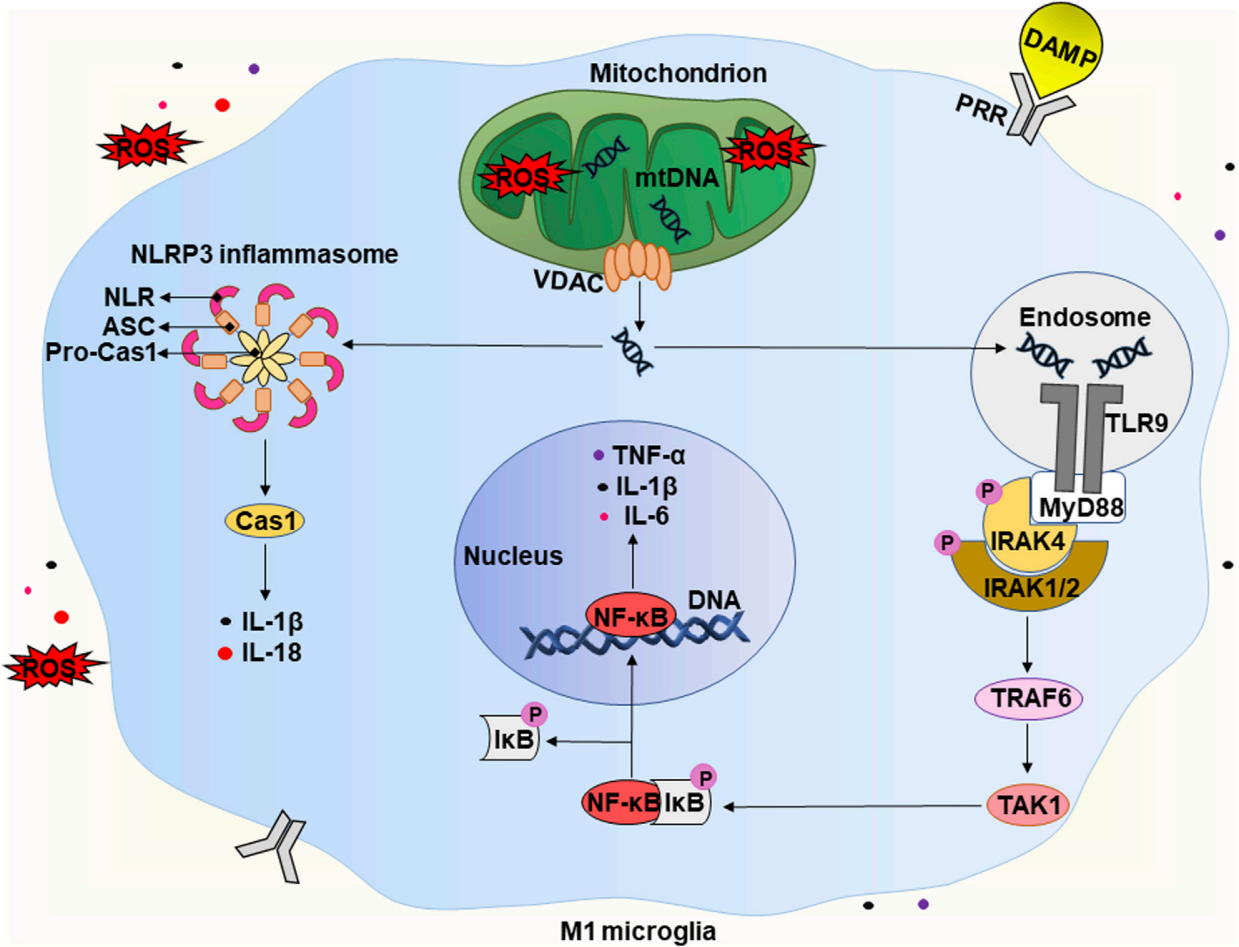
Potential mechanism of the development of “sterile” neuroinflammation in chronic vascular encephalopathy. A detailed description is given in the text. Abbreviations: DAMP – damage-associated molecular pattern, ROS – reactive oxygen species, mtDNA – mitochondrial deoxyribonucleic acid, VDAC – voltage-dependent anion channel, TLR9 – Toll-like receptor 9, PRR – pathogen recognition receptor, MyD88 – myeloid differentiation primary response 88, P – phosphate residue, IRAK1/2/4 – interleukin 1 receptor-associated kinase 1/2/4, TRAF6 – tumour necrosis factor receptor-associated factor 6, TAK1 – TGFβ activated kinase 1, IκB – inhibitor of kappa B, NF-κB – nuclear factor kappa B, IL-1β/6/18 – interleukins IL-1β/6/18, respectively, TNF-α – tumor necrosis factor α, NLRP3 – NLR family pyrin domain containing 3, ASC – apoptosis-associated speck-like protein containing a caspase recruitment domain (CARD), Pro-Cas1 – pro-caspase 1, Cas1 – caspase 1.

Microglia play a central role in the development of neuroinflammation. They make up about 10%–20% of the cell population of the CNS and provide primary immune surveillance. In a healthy brain under physiological conditions, microglial cells show the so-called “resting” morphology, that is characterized by a small cell body with numerous long, thin and highly branched processes. The latter are constantly moving, checking the surrounding space for signs of pathogen- or damage-related molecules, in particular, PAMPs and DAMPs (DiSabato et al., 2016; Raffaele and Fumagalli, 2022; Garaschuk and Verkhratsky, 2024). Transcriptome analysis of “resting” microglia shows that they significantly express genes associated with steady-state brain functions, including maintenance of homeostasis, neuronal maturation, and synaptic integrity. However, recognition of DAMPs activates microglial cells, changing their morphology and transcriptional profile (Wendimu and Hooks, 2022). Below we consider the details of the potential mechanisms of the development of the so-called “sterile” neuroinflammation in CVE (Figure 3).

As mentioned in Section 2 above, CVE is often accompanied by acute and chronic hypoxia that can lead to increased levels of reducing equivalents and ROS, causing the development of reductive and oxidative stress, respectively (Figure 2). The latter have various consequences for cells, including the release of endogenous DAMPs. Under physiological conditions, limited production of ROS is necessary for maintaining the biological functions of cells. However, at high concentrations, ROS are recognized as DAMPs and activate the innate immune system of the brain (Koenig and Buskiewicz-Koenig, 2022). In addition, the increase in ROS levels can overwhelm defense systems causing numerous oxidative modifications of biomolecules and contributing to the increase of other types of DAMPs. It is worth mentioning that mtDNA is the first and closest target for the harmful effects of mtROS (Roh and Sohn, 2018; Dmytriv et al., 2023). An increase in the level of mtROS leads to mitochondrial dysfunction, which is characterized by swelling of these intracellular organelles, an increase in inner membrane permeability, and a release of mtDNA (Figure 3) (Lin et al., 2022). The latter occurs with the participation of voltage-dependent anion channels (VDACs). This is the most common protein of the outer mitochondrial membrane and plays the role of a channel to transport ATP, Ca^2+^, and other metabolites between mitochondria and cytosol (Kmita et al., 2023). However, under stressful conditions, VDACs oligomerize to form macropores that provide the release of mtDNA (Kim et al., 2023).

The release of endogenous DAMPs, including mtDNA, contributes to the development of “sterile” inflammation that is not associated with pathogens. The brain contains a significant number of DAMP sensors, in particular, pathogen recognition receptors (PRRs). There are several subfamilies of PRRs, including Toll-like receptors (TLRs), C-type lectin receptors (CLRs), RIG-like receptors (RLRs), and Nod-like receptors (NLRs). RLRs and NLRs are expressed inside cells and recognize intracellular inflammation-related molecules. Generally, CLR and TLR are transmembrane receptors that contribute to the surveillance of the extracellular environment (Kigerl et al., 2014). However, there are some exceptions. For example, TLR9 is expressed intracellularly on the endoplasmic reticulum and under certain conditions migrates to endosomal membranes (De Gaetano et al., 2021). Thus, microglial cells can recognize DAMPs both intracellularly and extracellularly. This interaction activates receptors and morphologically transforms the branched state of these cells into an amoeboid state with a large cell body and almost no processes (Figure 1) (Wendimu and Hooks, 2022). Below, we take a closer look at the molecular mechanisms involved in the initiation of a pro-inflammatory response due to the release of mtDNA.

Released mtDNA is recognized by several intracellular receptors, for example, TLR9 and NLR (Figure 3). TLR9 interacts with mtDNA with a 2:2 stoichiometry (2 TLR9 monomers and two mtDNA molecules). This interaction leads to the activation of the first downstream target, the adapter protein MyD88 (myeloid differentiation primary response 88) (De Gaetano et al., 2021). The latter forms a complex with IRAK4 (interleukin one receptor-associated kinase 4), that recruits IRAK1 and IRAK2 resulting in their phosphorylation. Phosphorylated IRAKs interact with TRAF6 (tumor necrosis factor receptor-associated factor 6), that activats TAK-1 (TGFβ activated kinase 1) to mediate activation of the transcription factor NF-κB (Zheng et al., 2020). Under non-stress conditions, NF-κB is in a complex with IκB (inhibitor of NF-κB) and remains in the cytoplasm. Phosphorylation of IκB by the IKK complex (IκB kinase) leads to polyubiquitination of IκB with its subsequent proteasomal degradation (Christian et al., 2016). TAK1 also carries out an alternative phosphorylation of IκB, promoting the release of NF-κB. The latter translocates into the nucleus and activates the transcription of pro-inflammatory cytokines, in particular, IL-1β, IL-6, and TNF-α (Zheng et al., 2020.

In addition, some NLRs, when recognizing DAMPs, form a multi-protein complex called the “inflammasome”. It consists of NLR, an adapter protein (ASC), and a precursor of caspase 1 (pro-caspase 1). Assembly of the inflammasome triggers the autocleavage of pro-caspase one and the formation of functionally active caspase one that then cleaves pro-IL-1β and pro-IL-18 into their active forms (IL-1β and IL-18). The release of pro-inflammatory cytokines activates the pro-inflammatory microglial phenotype, that is called M1 (Galizzi and Di Carlo, 2023). M1 microglia actively produce IL-1β, IL-6, TNF-α, chemokines, and others. They also significantly expresses the enzyme NADPH oxidase, that produces ROS, in particular, the superoxide anion radical, and inducible nitric oxide synthase (iNOS) producing ^•^NO (Guo et al., 2022). In this way, microglial cells can damage nearby neurons and astrocytes. Potentially, this can lead to cognitive decline and brain damage, which are observed in patients with CVE. Production of pro-inflammatory cytokines and chemokines promotes the recruitment of peripheral immune cells, including monocytes and macrophages. These events initiate a number of pathophysiological processes, including both the production of pro-inflammatory cytokines and tissue damage, and the production of anti-inflammatory cytokines and healing. As a result, this can lead to demyelination, loss of synapses and death of neurons (Ising and Heneka, 2018; Fard and Stough, 2019).

Synapses are sites of cell–cell contact that transmit signals between neurons. They provide a molecular mechanism by which information is encoded, processed, and stored within the brain (Bailey and Kandel, 2008). Until recently, synapses were considered as simple connections between neurons. However, disruption of these connections causes significant violations of behavior, cognition, and memory (Adams et al., 2023). Loss of synapses is often a consequence of inflammation and has been found in many neurodegenerative diseases. It correlates with the severity of cognitive decline and contributes to cognitive impairment even in the absence of gross atrophy (Griffiths and Grant, 2023). Thus, even a small loss of synapses caused by the development of neuroinflammation in CVE can potentially lead to cognitive decline and subsequent dementia.

It is also worth mentioning the role of astrocytes, the most abundant type of glial cells in the CNS. Under homeostatic conditions, astrocytes provide baseline trophic support for neurons, coordinate the formation and functioning of synapses, and participate in forming the BBB. In addition, they are also immunocompetent cells that can recognize danger signals in their extracellular environment (Colombo and Farina, 2016). In particular, the release of pro-inflammatory mediators and ROS by microglial cells can cause a change in the morphological and functional characteristics of astrocytes to adopt a so-called “reactive” phenotype. Unlike typical astrocytes, that promote neuronal survival, reactive astrocytes downregulate accessory functions and begin to secrete neurotoxic factors, including complement components and chemokines, that promote the recruitment of immune cells across the BBB into the CNS (Lawrence et al., 2023). Potentially, this can lead to dysfunction of cerebral blood vessels, neurotoxicity, and the development of CVE. Reactive astrocytes proliferate, become hypertrophied, and increase the expression of intermediate filament proteins, cytokines, and chemokines. They are grouped into polarized bundles and connect with components of the extracellular matrix, forming astrocytic scars. The latter forms a physical barrier limiting inflammation (Cekanaviciute and Buckwalter, 2016). However, as far as we know, there is currently no information on astrocytic scars in patients with CVE.

## 4 Therapeutic approaches for the treatment of chronic vascular encephalopathy

Known therapeutic approaches for treating CVE face many obstacles, including polypharmacy, disease specificity, and complications such as neuroinflammation, excessive BBB permeability, ionic imbalance, etc. It should be noted that strategies for CVE treatment mostly focus on controlling its symptoms as well as the underlying cardiovascular and cerebrovascular risk factors (O'Brien et al., 2017). Next, we will consider several therapeutic and nutraceutical interventions based on counteracting ionic imbalance, BBB dysfunction, and inflammation in CVE.

Chronic cerebral hypoperfusion, one of the main pathological changes occurring in vascular dementia, also disrupts ion homeostasis decreasing K^+^ and increasing Ca^2+^ and Na^+^ levels (Poh et al., 2022). Multiple ion transporters and their regulatory kinases play crucial roles in the reactive astrogliosis process, so the use of their pharmacological inhibitors represents promising drugs for CVE (Rahman et al., 2024). For instance, bumetanide, a specific inhibitor of Na-K-Cl cotransporter 1, attenuates chronic hypoperfusion-induced white matter damage and cognitive impairment in a mouse model of vascular cognitive impairment and dementia (Yu et al., 2018). Chronic administration of nimodipine, a central nervous system-selective dihydropyridine calcium channel blocking agent provides an effective preventive treatment for stroke and cognitive decline in cerebral small vessel disease (Yang et al., 2024).

Zhang et al. (2024) demonstrated that vascular cell adhesion molecule 1 (VCAM1) is involved in BBB dysfunction in chronic cerebral hypoperfusion. In particular, VCAM1 expression directly correlates with the severity of BBB impairment, whereas blocking VCAM1 reduces BBB leakage and protects white matter integrity. In such a way, the use of inhibitors of VCAM1 may offer a new therapeutic approach to CVE treatment.

An analgesic neurotropin alleviates cognitive impairment by inhibiting the TLR4/MyD88/NF-κB signaling pathway in a mouse model of vascular dementia. In particular, it promotes cerebral blood flow, weakens damage to the white matter, reduces the loss of neurons, and improves cognitive functions (Zou et al., 2023). Pharmacological inhibition of NLRP3 inflammasome also has therapeutic potential in this field (Xu et al., 2023). In addition, preclinical studies of rodents strongly suggest that intermittent fasting (a dietary pattern alternating between eating and fasting periods within a 24-h cycle) attenuates pathological mechanisms, including inflammation, involved in vascular cognitive impairment (Rajeev et al., 2024).

Various substances of natural origin can also be used for CVE treatment. For example, sulforaphane is a powerful activator of the nuclear factor erythroid 2-related factor 2 (Nrf2), which upregulates the expression of a number of genes coding antioxidant and anti-inflammatory proteins/enzymes (Lushchak, 2014). Mao et al. (2019) found that sulforaphane-mediated neuroprotection in a rat model of vascular cognitive impairment was associated with enhanced activation of Nrf2 and upregulation of heme oxygenase 1. Thus, the use of sulforaphane increases the antioxidant potential of cells, inhibiting the development of oxidative stress and neuroinflammation (Townsend and Johnson, 2016; Zhou et al., 2016; Subedi et al., 2019).

Vitamin D may also be promising for CVE treatment. The vitamin D receptor (VDR) is a DNA-binding transcription factor and a member of the nuclear steroid hormone receptor family. VDR signaling in microglia inhibits neuroinflammation, whereas VDR knockout promotes a proinflammatory phenotype characterized by the production of proinflammatory cytokines (Cui et al., 2023).

## 5 Conclusion and perspectives

**FIGURE 4:**
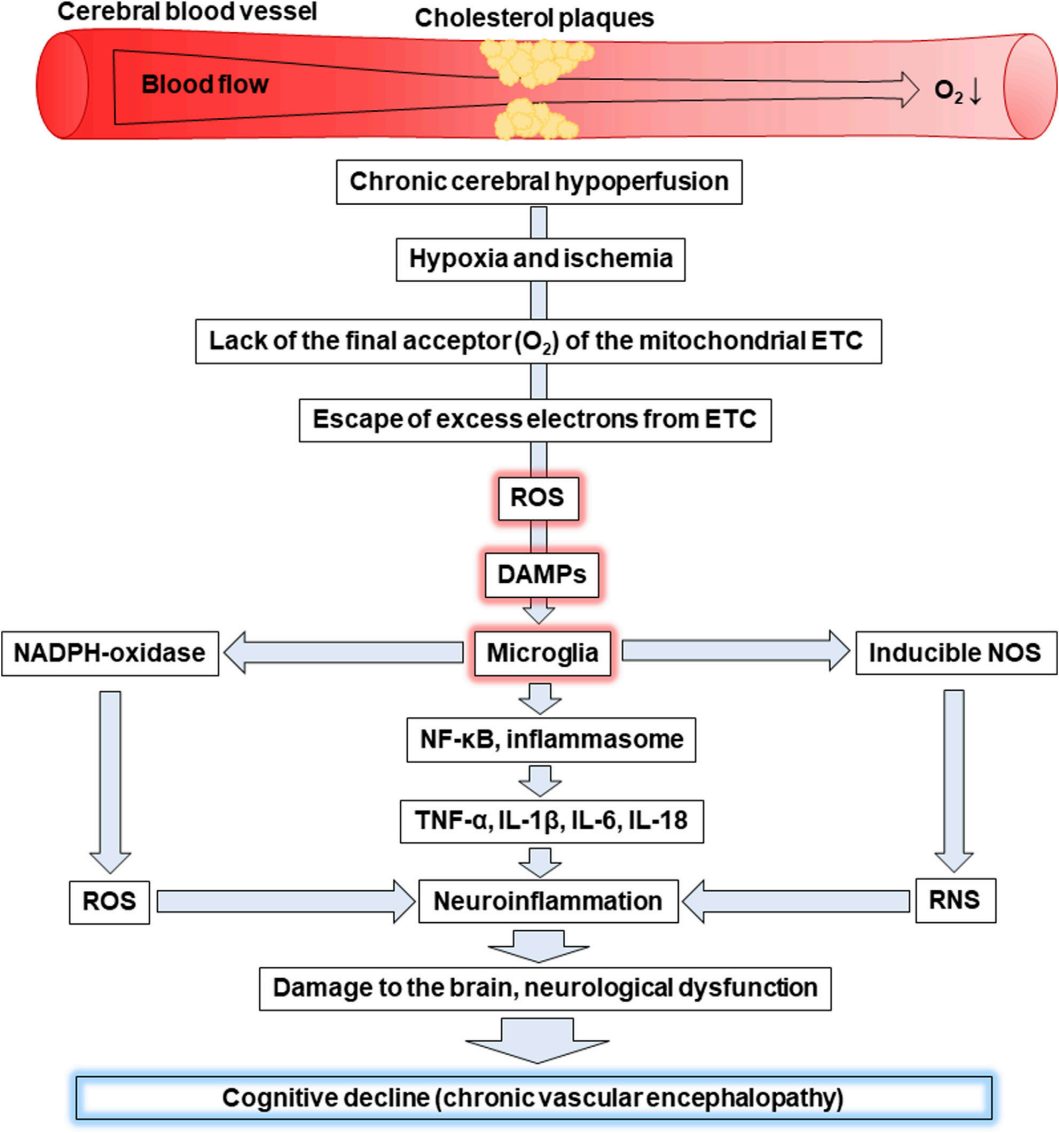
Cognitive decline as a result of hypoxia-induced increased levels of ROS, DAMPs, and activation of microglia. Abbreviations: ETC – electron-transport chain, ROS – reactive oxygen species, DAMPs – damage-associated molecular patterns, NOS – nitric oxide synthase, RNS – reactive nitrogen species, NF-κB - nuclear factor kappa B, TNF-α – tumour necrosis factor α, IL – interleukin.

CVE is a frequent cause of cognitive impairment, including mild vascular cognitive impairment and dementia. It occurs as a result of chronic disorders of the blood supply to the brain, in particular, chronic cerebral hypoperfusion, which causes hypoxia and ischemia. ROS and DAMPs are among the key players in the pathophysiology of CVE. Figure 4 shows a scheme that connects all of these concepts into one chain of sequential events that leads to the cognitive decline characteristic of CVE.

Chronic cerebral hypoperfusion is associated with insufficient blood supply to the brain. For example, the deposition of cholesterol plaques on the walls of cerebral vessels can cause reduced blood flow to the brain, leading to hypoxia and ischemia. Oxygen is the final acceptor of electrons in the mitochondrial ETC and its lack is primarily associated with a decrease in ATP production, as well as increased generation of ROS. The latter is a consequence of excess electrons escaping from the ETC causing the formation of ROS. This leads to the development of oxidative stress as witnessed by oxidative modifications of proteins, lipids, and nucleic acids.

Mitochondria, as the site of the ETC are the first target for the harmful effects of mtROS. As a result, they swell, become more permeable and release mtDNA, which together with mtROS are recognized as DAMPs. Microglial cells can face DAMPs released as a result of apoptosis or necrosis of neighbouring cells and recognize them through transmembrane PRRs or recognize their own endogenous DAMPs, including mtROS and mtDNA through intracellular PRRs, for example, TLR9. Oxidative stress can be the cause of both events. The interaction of DAMPs and PPRs leads to the activation of microglia characterized by enhanced expression of NADPH oxidase, which produces the superoxide anion radical, and inducible NOS, which generates ^•^NO. In general, this promotes an increase in ROS and RNS levels resulting in the induction of oxidative and nitrosative stress, respectively. In addition, activated microglia are associated with the activation of the transcription factor NF-κB and the inflammasome. Both contribute to the production of pro-inflammatory cytokines, including TNF-α, IL-1β, IL-6, IL-18 inducing neuroinflammation. In addition, microglia release chemokines that ensure the recruitment of peripheral leukocytes. In general, all of the events listed above can lead to neuronal degradation, demyelination, and loss of synapses causing the significant cognitive decline characteristic of CVE. Thus, oxidative stress caused by the increase of ROS and neuroinflammation caused by the release of DAMPs, which are recognized by microglia, play a crucial role in the progression of CVE.

Most neurodegenerative diseases are currently considered incurable, so the search for various methods for their prevention and treatment remains relevant. For example, the use of sulforaphane, an isothiocyanate of the cruciferous family, and vitamin D as dietary supplements may be promising for the relief of CVE symptoms. Potentially, they can be used to break the vicious cycle (Figure 1) of neurodegeneration associated with oxidative stress and neuroinflammation. For example, the inhibition of NLRP3, a key player in inflammation (Figure 3), slows neurodegeneration and alleviates cognitive impairment (Dempsey et al., 2017; Liang et al., 2022). In addition, a decrease in IL-1β production alleviates hypoperfusion-induced brain injury (Poh et al., 2021). Together, this indicates the essential role of neuroinflammation in the progression of CVE.

Sulforaphane inhibits neuroinflammation and associated oxidative stress. In particular, it activates the transcription factor Nrf2 and the expression of its target genes in microglia. The latter include NQO1 (NAD(P)H quinone dehydrogenase 1) and HMOX1 (heme oxygenase 1) (Townsend and Johnson, 2016). NQO1 catalyzes the two-electron reduction of quinones and many other compounds in the cytosol. This minimizes the formation of reactive semiquinones resulting from one-electron reduction as well as ROS (Dinkova-Kostova and Talalay, 2010). HMOX1 is the enzyme responsible for the degradation of heme into three products: carbon monoxide, iron ions, and biliverdin. The latter is successively transformed into a powerful antioxidant bilirubin by biliverdin reductase (Consoli et al., 2021). In addition, Nrf2 upregulates the expression of SOD1 (superoxide dismutase 1) (Daněk et al., 2022), that scavenges the superoxide anion radical leading to the formation of hydrogen peroxide, that is further neutralized by catalase and peroxidases (Bayliak et al., 2023). In general, the increase in antioxidant enzyme activities enhances the antioxidant potential and capability to degrade ROS. In turn, this suppresses neuroinflammation. In particular, sulforaphane decreases levels of pro-inflammatory cytokines, including IL-1β and IL-6, as well as reduces the expression of iNOS. This indicates that it is a potentially beneficial dietary supplement that can reduce microglia-mediated oxidative stress and neuroinflammation (Townsend and Johnson, 2016). In addition, increased expression of Nrf2 blocks the induction of astrocyte reactive gene expression by counteracting NF-κB, whereas the absence of Nrf2 conversely promotes the activation of reactive astrocytes. Thus, the use of Nrf2 activators such as sulforaphane can potentially prevent astrocyte reactivity, suppressing neuroinflammation (Nakano-Kobayashi et al., 2023).

Vitamin D can also be used in this direction. It downregulates the expression of NF-κB, a crucial neuroinflammatory factor (Figure 3), and markedly suppresses IL-6 and IL-17 levels (Hajiluian et al., 2017). In turn, this prevents excessive permeability of the BBB and cognitive dysfunction. Thus, both sulforaphane and vitamin D may be promising supplements for the prevention and treatment of CVE, particularly for breaking the vicious cycle of oxidative stress and neuroinflammation (Figure 1) that underlies neurodegeneration.

Taking into account all of the above, the following directions for future research in the field of CVE can be outlined:

1. Systemic investigation of the role of oxidative stress induced by hypoxia and reoxygenation in neurodegeneration.
2. Research on reductive stress under hypoxic conditions.
3. The use of sulforaphane and vitamin D for the treatment of CVE.
4. The potential of antioxidant therapy in patients with CVE.
